# Characterization of a newly isolated broad-host-range lytic phage WEN7 against multidrug-resistant clinical *Pseudomonas aeruginosa* strains

**DOI:** 10.3389/fmicb.2026.1861197

**Published:** 2026-08-12

**Authors:** Chang Wen, Xiaohong Xiao, Jinyi Chen, Xuan Zou, Shi Chen, Lianrong Wang

**Affiliations:** 1 Department of Gastroenterology, Hubei Clinical Center and Key Laboratory of Intestinal and Colorectal Disease, Ministry of Education Key Laboratory of Combinatorial Biosynthesis and Drug Discovery, Zhongnan Hospital of Wuhan University, School of Pharmaceutical Sciences, Wuhan University, Wuhan, China; 2 Department of Critical Care Medicine, Intensive Care Unit, Shenzhen Key Laboratory of Microbiology in Genomic Modification & Editing and Application, Shenzhen Institute of Translational Medicine, Shenzhen University Medical School, Shenzhen Second People’s Hospital, The First Affiliated Hospital of Shenzhen University, Shenzhen, China; 3 Department of Respiratory Diseases, Institute of Pediatrics, Shenzhen University Medical School, Shenzhen Children’s Hospital, Shenzhen, China

**Keywords:** bacteriophage therapy, broad host range, environmental stability, genome analysis, *Pseudomonas aeruginosa*

## Abstract

**Introduction:**

*Pseudomonas aeruginosa* is a Gram-negative opportunistic pathogen with intrinsic and acquired resistance to multiple classes of antibiotics. Phage therapy has emerged as a promising approach to combat multidrug-resistant bacterial infections.

**Methods:**

In this study, a lytic *P. aeruginosa* phage, WEN7, was isolated from hospital wastewater using PAO1-SZ1 as the host. We systematically characterized its morphology, growth kinetics, stability, host range, whole-genome sequence and bactericidal performance.

**Results:**

WEN7 formed clear plaques. Transmission electron microscopy revealed an icosahedral 66 nm head attached to a contractile tail, with intact virions captured in both fully extended (140 nm total tail length) and sheath-contracted (60 nm) states—this classic myoviral morphology places the phage within the class Caudoviricetes. One-step growth curve analysis revealed a latent period of approximately 25 min and an average burst size of about 165 PFU/cell. WEN7 remained stable under various temperatures, pH values, UV exposure, and ethanol concentrations. The phage displayed a broad host range, lysing 19 of the 29 clinical *P. aeruginosa* isolates tested (65.5%). Its genome is a circular double-stranded DNA molecule of 66,379 bp with a GC content of 55.62%. Notably, no known antibiotic resistance genes or virulence factors were annotated. WEN7 shares the highest genomic similarity with the *Pseudomonas* phage PCCM_PaP004 (genus *Pbunavirus*). At low multiplicities of infection, WEN7 showed strong bactericidal activity in both milk and hospital wastewater.

**Discussion:**

Together, these results indicate that phage WEN7 is a promising candidate for phage therapy against multidrug-resistant *P. aeruginosa* infections.

## 1 Introduction

*Pseudomonas aeruginosa* is a Gram-negative opportunistic pathogen that inhabits natural environments such as sewage and soil (Diggle and Whiteley, 2020). It can also colonize human skin, the external ear canal, and the respiratory tract (Qin et al., 2022). Severe healthcare-associated infections frequently involve this pathogen, manifesting as respiratory tract infections, ventilator-associated pneumonia, and catheter-related bacteremia (Mesas Vaz et al., 2025). In patients with cystic fibrosis, *P. aeruginosa* readily forms stable biofilms in the lungs (Malhotra et al., 2019), leading to chronic colonization that is difficult to eradicate. Carbapenems and third-generation cephalosporins are commonly used to treat bacterial infections (Solomon and Oliver, 2014). However, *P. aeruginosa* exhibits intrinsic resistance to carbapenems and other key antibiotics, largely due to mutations in the outer membrane porin OprD and overexpression of AmpC *β*-lactamase; biofilm-associated extracellular polysaccharides further impede antibiotic penetration (Angeletti et al., 2018).

Widespread overuse of antibiotics has driven a rapid increase in antimicrobial resistance worldwide (Abbas et al., 2024). A 2019 study in *The Lancet* estimated that approximately 1.27 million deaths resulted from infections caused by drug-resistant bacteria that year (Antimicrobial Resistance Collaborators, 2022). By 2024, a follow-up study projected that antimicrobial resistance could cause more than 39 million cumulative deaths between 2025 and 2050 (GBD 2021 Antimicrobial Resistance Collaborators, 2024). *P. aeruginosa* is a prime example of such resistant pathogens. Multidrug-resistant isolates have become increasingly common, with resistance levels continuing to rise. In a study from Guangdong, China, the prevalence of carbapenem resistance in *P. aeruginosa* remained around 24% during 2022-2023 (Li et al., 2025). More recent data indicate that the global average resistance rate of *P. aeruginosa* to meropenem has reached 23.3% and continues to climb (Ahmadi et al., 2025). Given this situation, exploring alternative strategies beyond conventional antibiotics has become increasingly urgent.

Before the introduction of penicillin into clinical practice, phage therapy was a mainstream antibacterial approach. Its use declined with the rise of broad-spectrum antibiotics, but recent concerns over antimicrobial resistance have revived interest in phage therapy (Strathdee et al., 2023). Unlike antibiotics, phages are highly specific, can self-amplify at infection sites, and disrupt biofilms through enzymes such as depolymerases (Roach and Donovan, 2015). Clinical studies have reported promising results, with improvement rates around 78.8%, bacterial clearance up to 86.7%, and few side effects (Uyttebroek et al., 2022). For *P. aeruginosa* infections, Ferry et al. (2022) successfully treated a patient with a pandrug-resistant spinal infection using phages, and Aslam et al. (2019) cured two cases of drug-resistant pneumonia with a phage cocktail. Beyond these individual cases, phage-antibiotic synergy (PAS) offers advantages against multidrug-resistant infections, enhancing bacterial killing and slowing the emergence of resistance. This combined strategy is currently being tested in clinical settings (Pirnay et al., 2024).

Nevertheless, phage therapy still faces numerous challenges. During long-term co-evolution, bacteria have developed diverse antiviral defense systems, including the DNA phosphorothioation (PT) modification system (Wang et al., 2007; Xiong et al., 2020; Wu et al., 2022; An et al., 2025; Wang et al., 2024) and the Ppl defense system (Xu et al., 2026), which are the focus of our laboratory’s research. Notably, these systems may also be present in clinically isolated multidrug-resistant strains, potentially contributing to the failure of phage therapy. Fortunately, phages have evolved corresponding escape strategies, such as genome modification (He et al., 2026; Wang et al., 2026) or encoding kinases (Jiang et al., 2024), to circumvent host defenses. Another challenge is the limited diversity of phages available for clinical use (Pirnay et al., 2024). Although phages are abundant in nature, few have been developed for therapeutic applications (Djebara et al., 2025). Therefore, isolating novel phages from the environment remains an important way forward. However, isolation alone is insufficient; successful translation of phage therapy into clinical practice also depends on robust phage performance under diverse environmental conditions. Moreover, *P. aeruginosa* is not only a significant nosocomial pathogen but also a common contaminant in food products and a major constituent of hospital wastewater, which serves as a reservoir for the spread of multidrug-resistant strains. Therefore, evaluating phage bactericidal activity in milk and hospital wastewater provides insights into its potential for both food safety applications and environmental disinfection, in addition to its clinical promise. These two matrices were selected to represent nutrient-rich and complex chemical environments, respectively, reflecting realistic scenarios where phages might be deployed in various application contexts.

In this study, we isolated a *P. aeruginosa* phage, designated WEN7, from hospital wastewater. We characterized its biological properties, genome features and phylogenetic relationships, and tested its bactericidal efficacy in milk and in hospital wastewater. This study expands the repertoire of phages available against multidrug-resistant *P. aeruginosa* and provides a foundation for future therapeutic development.

## 2 Materials and methods

### 2.1 Bacterial strains and culture conditions

A total of 36 bacterial strains were used in this study. *Pseudomonas aeruginosa* PAO1-SZ1, a multidrug-resistant derivative of the model strain PAO1, served as the host strain for phage isolation. Table 1 presents the antibiotic resistance profile of this host against a panel of 10 antibiotics. The 29 clinical *P. aeruginosa* isolates were obtained from inpatients at Shenzhen Second People’s Hospital (antibiotic resistance profiles are provided in Supplementary Table S1). The six reference strains from our laboratory collection: *Pseudomonas* sp. XWY-1 (GenBank: NZ_CP026332), *P. aeruginosa* MPAO1 (GenBank: CP116237.1), *P. putida* KT2440 (GenBank: NC_002947.4), *P. fluorescens* Pf0-1 (GenBank: CP000094.2), *Bacillus subtilis* 168 (GenBank: NC_000964.3), and *B. thuringiensis* CTC (GenBank: CP013273.1). All strains were incubated at 37 °C in Luria-Bertani (LB) broth with shaking at 220 rpm or on LB agar plates.

**Table 1:** Antibiotic susceptibility profile of *Pseudomonas aeruginosa* PAO1-SZ1.

Antibiotic	Final concentration (μg/mL)	Susceptibility
Nalidixic acid	25	R
Ampicillin	100	R
Chloramphenicol	25	R
Kanamycin	50	R
Apramycin	25	R
Thiamphenicol	15	R
Gentamicin	50	S
Rifampicin	50	S
D-Cycloserine	250	R
Streptomycin	50	R

S, susceptible; R, resistant.

### 2.2 Phage isolation, purification, and propagation

Phage WEN7 was recovered from hospital wastewater. Briefly, 60 mL of 3 × LB broth, 15 mL of host bacterial culture in logarithmic phase (OD_600_ = 0.6), and 60 mL of wastewater were mixed and incubated overnight at 37 °C with shaking at 220 rpm. The next day, 2 mL of the mixture was centrifuged at 12,000 rpm for 3 min, and the supernatant was filtered through a sterile 0.22-μm membrane. For initial screening, 100 μL of the filtrate was mixed with 200 μL of host culture and 5 mL of molten LB soft agar (0.75%, w/v), poured over LB agar plates (1.5%, w/v), and incubated overnight at 37 °C. Multiple plaque morphologies were observed during primary screening; only large, fully clear plaques with regular morphology were selected for further purification, as clear plaques indicate strong lytic activity. A single well-defined plaque was picked and successively streaked onto fresh host-seeded LB agar plates. After three to five rounds of single-plaque purification, a pure phage isolate was obtained. For high-titer stock preparation, 2 mL of host suspension was incubated with 50 mL of fresh LB broth overnight at 37 °C. The lysate was divided into 1 mL aliquots and stored at 4 °C for short-term use or mixed with sterile glycerol (final concentration 20%) and kept at −80 °C for long-term preservation.

Phage titers were determined by spot assay on double-layer agar plates (Paranos et al., 2024). Briefly, double-layer agar plates were prepared with a bottom layer of LB agar (1.5%, w/v) and a top layer of LB soft agar (0.75%, w/v) mixed with 200 μL of overnight host culture. Phage lysates were serially diluted in SM buffer (100 mM NaCl, 8 mM MgSO_4_, 50 mM Tris–HCl, pH 7.5) (Huang et al., 2025), and 3 μL aliquots of each 10-fold dilution were spotted onto the bacterial lawn. After overnight incubation at 37 °C, plaques were counted at the highest countable dilution. The titer was calculated as: (number of plaques × dilution factor × 1,000)/3, where the factor × 1,000 converts the 3 μL spotting volume to 1 mL for the final titer expressed as PFU/mL.

### 2.3 Determination of host range

The host range of phage WEN7 was assessed against the 29 clinical *P. aeruginosa* isolates and six reference strains listed in Section 2.1. A 10 μL aliquot of the phage stock (approximately 10^8^ PFU/mL) was spotted onto a lawn of each test strain, and the plates were incubated overnight at 37 °C. Lysis was scored as: +++, complete; ++, turbid; +, weak; −, no lysis.

### 2.4 Transmission electron microscopy

The morphology of phage WEN7 was examined using a Hitachi HT7700 transmission electron microscope (TEM) operating at 80 kV. 20 μL of phage stock (approximately 10^9^ PFU/mL) was placed onto a 200 mesh copper grid and left for 5 min. The grid was then negatively stained with 1% phosphotungstic acid for 10 min. Excess stain was removed with filter paper, and the grid was air-dried before imaging (Ackermann, 2009).

### 2.5 Optimal multiplicity of infection and one-step growth curve

To determine the optimal multiplicity of infection (MOI), *P. aeruginosa* PAO1-SZ1 was grown to exponential phase (approximately 10^8^ CFU/mL), Phage WEN7 was serially diluted to achieve MOIs of 0.001, 0.01, 0.1, 1, 10, 100, and 1,000. For each MOI, 100 μL of bacterial suspension was mixed with 100 μL of the corresponding phage dilution in 1.8 mL of LB broth and incubated overnight at 37 °C with shaking at 220 rpm. The culture was then filtered through a 0.22-μm filter, and the phage titer in the filtrate was measured by spot assay on double-layer agar plates. The MOI yielding the highest phage titer was taken as the optimal MOI. All assays were performed in triplicate.

A one-step growth curve was then performed at this optimal MOI. Briefly, 200 μL of bacterial culture and 200 μL of phage WEN7 were added to 19.6 mL of LB broth and incubated at 37 °C with shaking at 220 rpm for 15 min. The mixture was centrifuged at 8,000 rpm for 5 min, and the supernatant was discarded to remove unadsorbed phages (Pajunen et al., 2000). The pellet was resuspended in 20 mL of LB, centrifuged again under the same conditions, and the supernatant was removed once more. The final pellet was resuspended in 20 mL of sterile LB and incubated at 37 °C with shaking at 220 rpm for 2 h. At each time point, 500 μL samples were collected at 5 min intervals for the first 30 min and at 10 min intervals thereafter. Each sample was immediately filtered through a 0.22‑μm filter, and the phage titer was determined by spot assay on double-layer agar plates. All assays were performed in triplicate.

### 2.6 Stability analysis

A high-titer stock (approximately 10^8^ PFU/mL) of phage WEN7 was used for all stability assays (Liu et al., 2026). For thermal stability, 500 μL aliquots of the phage stock were incubated in water baths set to 4, 25, 30, 40, 50, 60, 70, and 80 °C for 1 h. For UV sensitivity, 1 mL aliquots of the phage stock were placed in a sterile Petri dish and exposed to UV light (254 nm, 15 W) on the work surface of a biosafety cabinet at a fixed irradiation distance of 30 cm for up to 120 min. Samples were collected at intervals of 0, 3, 6, 9, 12, 15, 30, 40, 50, 60, 70, 80, 90, 100, 110, and 120 min. For pH tolerance, LB broth was adjusted to pH values ranging from 3.0 to 11.0 (in increments of 1.0 pH unit) using 1 M HCl or 1 M NaOH, filter-sterilized through a 0.22‑μm membrane, and dispensed into sterile tubes. Then, 100 μL of the phage stock was mixed with 900 μL of each pH-adjusted LB broth and incubated at 30 °C for 1 h. For ethanol sensitivity, the phage stock was mixed with absolute ethanol to final concentrations of 25, 50, 75, and 99% (v/v), and incubated at 30 °C for 1 h. After each treatment, samples were immediately serially diluted in SM buffer (pH 7.0), and titers were determined by spot assay on double-layer agar plates. All assays were performed in triplicate.

### 2.7 Genome sequencing and analysis

Purified phage WEN7 was treated with DNase I and RNase A (final concentration 1 μg/mL each) at 37 °C for 2 h to remove host nucleic acid contaminants. The phage particles were then concentrated by PEG precipitation (10% PEG 8000, incubated on ice for 1 h followed by centrifugation) (Sarkar et al., 2026), and genomic DNA was extracted using a commercial kit. Whole-genome sequencing was performed by Nextomics Biosciences Co., Ltd. (Wuhan, China) on an Illumina platform. Assembly was carried out with SPAdes (Bankevich et al., 2012), and assembly quality was assessed with CheckM (Parks et al., 2015). Gene prediction was performed using Prodigal (Hyatt et al., 2010) and Prokka (Seemann, 2014). Genomic islands, prophages and CRISPR sequences were identified with Alien_Hunter (Langille et al., 2010) and PhiSpy (Akhter et al., 2012). Antibiotic resistance genes were predicted using the RGI tool against the CARD database (Alcock et al., 2020), and virulence factor genes were identified with Abricate against the VFDB database (Liu et al., 2019). A neighbor-joining phylogenetic tree was constructed in MEGA12 (Kumar et al., 2024) with 1,000 bootstrap replicates. Average nucleotide identity (ANI) was calculated with taxMyPhage (Millard et al., 2025) to evaluate the taxonomic status of phage WEN7. Genome collinearity analysis was performed with Easyfig (Sullivan et al., 2011). A circular genome map was generated using Proksee (Grant et al., 2023). The complete genome sequence and annotation were submitted to NCBI to obtain a GenBank accession number.

### 2.8 Lytic activity assay

To assess the lytic activity of phage WEN7 against its host *P. aeruginosa* PAO1-SZ1, bacterial killing assays were performed in 96-well plates (Rieper et al., 2025). A concentrated phage stock (10^11^ PFU/mL) was prepared separately and serially diluted in LB broth to titers of 10^10^, 10^9^, 10^8^, 10^7^, 10^6^, and 10^5^ PFU/mL. For each dilution, 2 μL of mid-log phase host culture (10^8^ CFU/mL) was mixed with 2 μL of the diluted phage and 196 μL of LB broth to achieve different MOIs. The mixtures were incubated at 37 °C with shaking, and the optical density at 600 nm (OD_600_) was measured every 30 min using a microplate reader. Bacterial culture without phage served as the negative control. All assays were performed in triplicate.

### 2.9 Application in milk and wastewater

Milk and hospital wastewater were selected as testing matrices to evaluate phage bactericidal activity in food-related and environmentally relevant contexts, respectively, reflecting potential application scenarios for biocontrol and phage therapy (Yang et al., 2025; Liu et al., 2026). For assays in pasteurized milk, 200 μL of host strain culture (10^8^ CFU/mL) and 200 μL of phage WEN7 (10^5^–10^11^ PFU/mL) were added to 19.6 mL of pasteurized milk (final volume: 20 mL) to achieve MOIs ranging from 0.001 to 1,000; a phage-free control was included. The mixtures were incubated overnight at 37 °C with shaking. For hospital wastewater assays, samples were collected from Shenzhen Second People’s Hospital, Shenzhen Children’s Hospital, and the Shenzhen Institute of Translational Medicine. Each sample was centrifuged at 6,000 rpm for 6 min and filtered through a 0.22‑μm filter to eliminate background microbial populations, enabling a controlled assessment of phage bactericidal activity against the target host in a natural chemical background. Then, 19.6 mL of sterile wastewater was mixed with the host strain and phage WEN7 (10^5^–10^11^ PFU/mL) at the same MOIs as above and incubated under the same conditions. After incubation, viable bacterial counts were determined by serial dilution of the co-culture mixtures and spotting 3 μL aliquots onto LB agar plates in triplicate, followed by overnight incubation at 37 °C. All assays were performed in triplicate.

## 3 Results

### 3.1 Phage isolation and morphology

**Figure 1:**
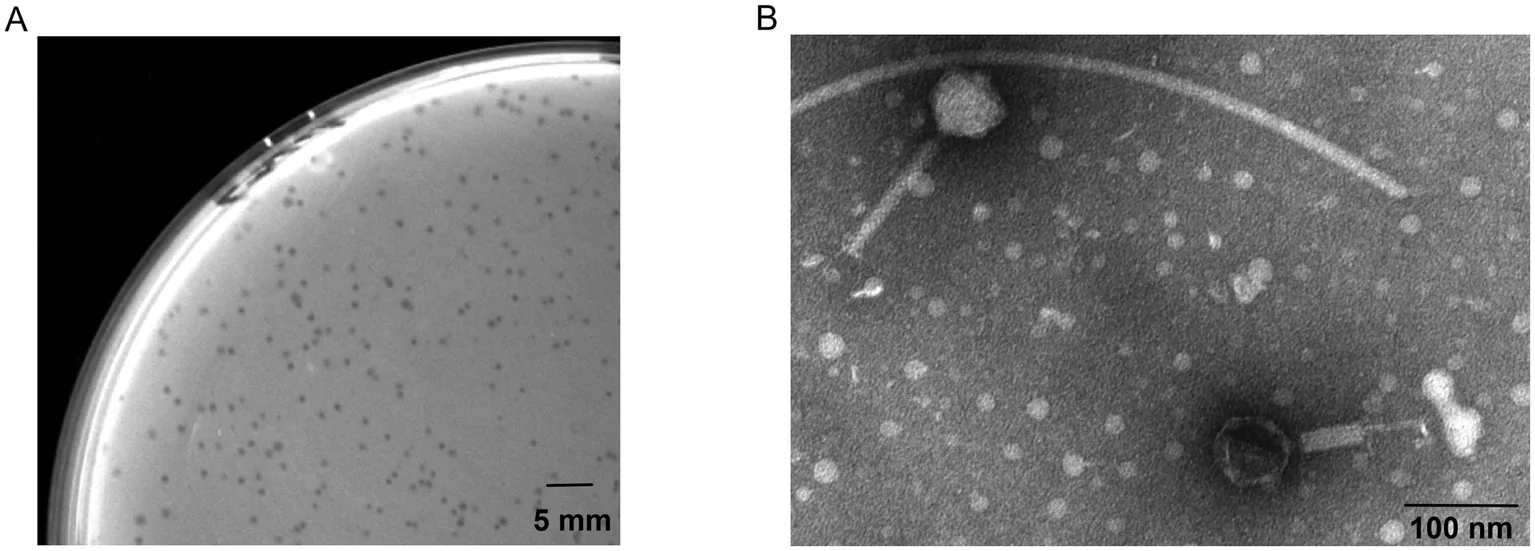
Morphology of phage WEN7. **(A)** Phage WEN7 formed clear, round plaques approximately 1 mm in diameter on host lawns. **(B)** Transmission electron microscopy (TEM) micrograph of phage WEN7. Scale bar: 100 nm.

Phage WEN7, a newly isolated *P. aeruginosa* phage, was obtained from hospital wastewater collected at the sewage treatment center of Shenzhen Second People’s Hospital, China. On the host lawn, WEN7 formed clear, round plaques approximately 1 mm in diameter (Figure 1A). Transmission electron microscopy revealed an icosahedral head of about 66 nm in diameter. Two distinct tail states were observed in the same image: one with an intact, extended tail approximately 140 nm long, and another with a contracted tail sheath of about 60 nm (Figure 1B). According to the current International Committee on Taxonomy of Viruses (ICTV) taxonomy, phage WEN7 belongs to the class Caudoviricetes (Turner et al., 2023).

### 3.2 Host range and antibiotic resistance

The antibiotic susceptibility of the host *P. aeruginosa* PAO1-SZ1 was tested against 10 antibiotics. PAO1-SZ1 was resistant to eight of them (Table 1), confirming its status as a multidrug-resistant strain. To assess the host range of phage WEN7, we tested 29 clinical multidrug-resistant *P. aeruginosa* isolates and six reference strains. Phage WEN7 lysed 19 of the clinical isolates and the model strain *P. aeruginosa* MPAO1, albeit with varying efficiencies (Table 2). No lysis was observed against any of the five non-*P. aeruginosa* reference strains tested, including representative species such as *B. subtilis* 168 and *P. putida* KT2440 (Supplementary Figure 1). These results indicate that phage WEN7 has a broad host range specifically targeting multidrug-resistant *P. aeruginosa*. Its inability to infect other bacterial species further highlights its potential for biocontrol applications.

**Table 2:** Bacterial strains used for host range test.

Bacterial species	Strains	No. of resistant antibiotics tested	Lysis	Source
*Pseudomonas aeruginosa*	PA3	2	+++	Clinical isolates
	PA5	2	+++	Clinical isolates
	PA6	2	+++	Clinical isolates
	PA8	2	+++	Clinical isolates
	PA13	2	+++	Clinical isolates
	PA15	1	+++	Clinical isolates
	PA23	8	+++	Clinical isolates
	PA24	8	+++	Clinical isolates
	PA25	3	+++	Clinical isolates
	PA26	6	+++	Clinical isolates
	PA28	2	+++	Clinical isolates
	PA30	7	+++	Clinical isolates
	PA4	3	++	Clinical isolates
	PA11	2	++	Clinical isolates
	PA27	1	++	Clinical isolates
	PA19	2	+	Clinical isolates
	PA20	2	+	Clinical isolates
	PA21	2	+	Clinical isolates
	PA29	9	+	Clinical isolates
	PA1	0	−	Clinical isolates
	PA7	1	−	Clinical isolates
	PA9	0	−	Clinical isolates
	PA10	1	−	Clinical isolates
	PA12	1	−	Clinical isolates
	PA14	2	−	Clinical isolates
	PA16	2	−	Clinical isolates
	PA17	2	−	Clinical isolates
	PA18	2	−	Clinical isolates
	PA22	2	−	Clinical isolates
	MPAO1	N/A	+++	Lab strains
*Bacillus subtilis*	168	N/A	−	Lab strains
*Bacillus thuringiensis*	CTC	N/A	−	Lab strains
*Pseudomonas putida*	KT2440	N/A	−	Lab strains
*Pseudomonas fluorescens*	Pf0-1	N/A	−	Lab strains
*Pseudomonas* sp.	XWY-1	N/A	−	Lab strains

Resistance count is shown only for clinical isolates (tested against up to 15 antibiotics; see Supplementary Table S1 for complete profiles). N/A, not applicable (laboratory strains, not tested). Lysis: +++, complete clearing; ++, turbid; +, weak; −, no lysis.

### 3.3 Optimal MOI and one-step growth curve

**Figure 2:**
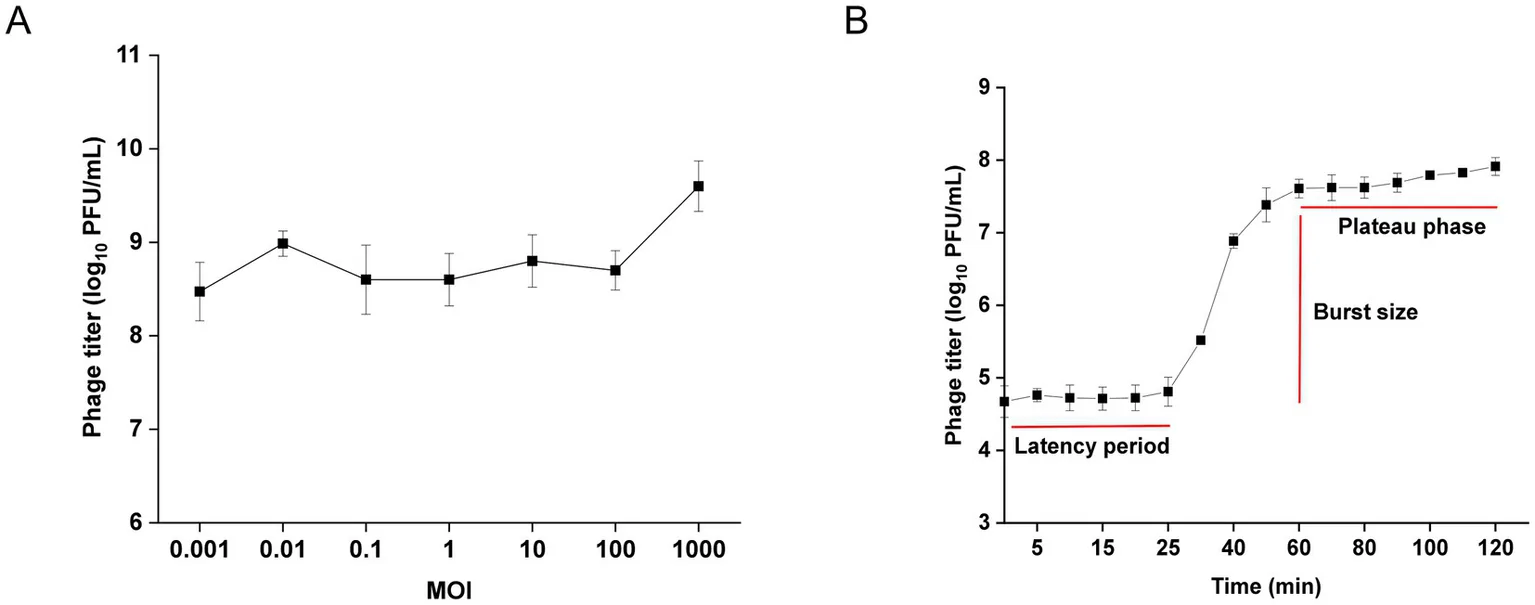
Optimal MOI and one-step growth curve of phage WEN7. **(A)** Phage titers obtained after 24 h of incubation at different MOIs in *P. aeruginosa* PAO1-SZ1 culture. The highest titer was observed at MOI = 1,000. **(B)** One-step growth curve at MOI = 1,000. The latent period, burst size and plateau phase are labeled. Error bars represent SD from three independent experiments.

To determine the optimal multiplicity of infection (MOI) for phage WEN7, its lytic activity was assessed at MOIs of 0.001, 0.01, 0.1, 1, 10, 100, and 1,000. Phage WEN7 maintained relatively high titers across all MOIs tested, with only minor differences. The highest titer was observed at an MOI of 1,000, which was therefore taken as the optimal MOI (Figure 2A).
A one-step growth curve revealed a latent period of 25–30 min, during which no significant increase in titer was detected (approximately 10^5^ PFU/mL). A rapid lytic phase followed from 30 to 60 min, after which the titer plateaued at around 10^8^ PFU/mL (Figure 2B). The burst size was estimated at approximately 165 PFU/cell, calculated as the net increase in total phage particles divided by the initial number of infected cells.

### 3.4 Phage stability

**Figure 3:**
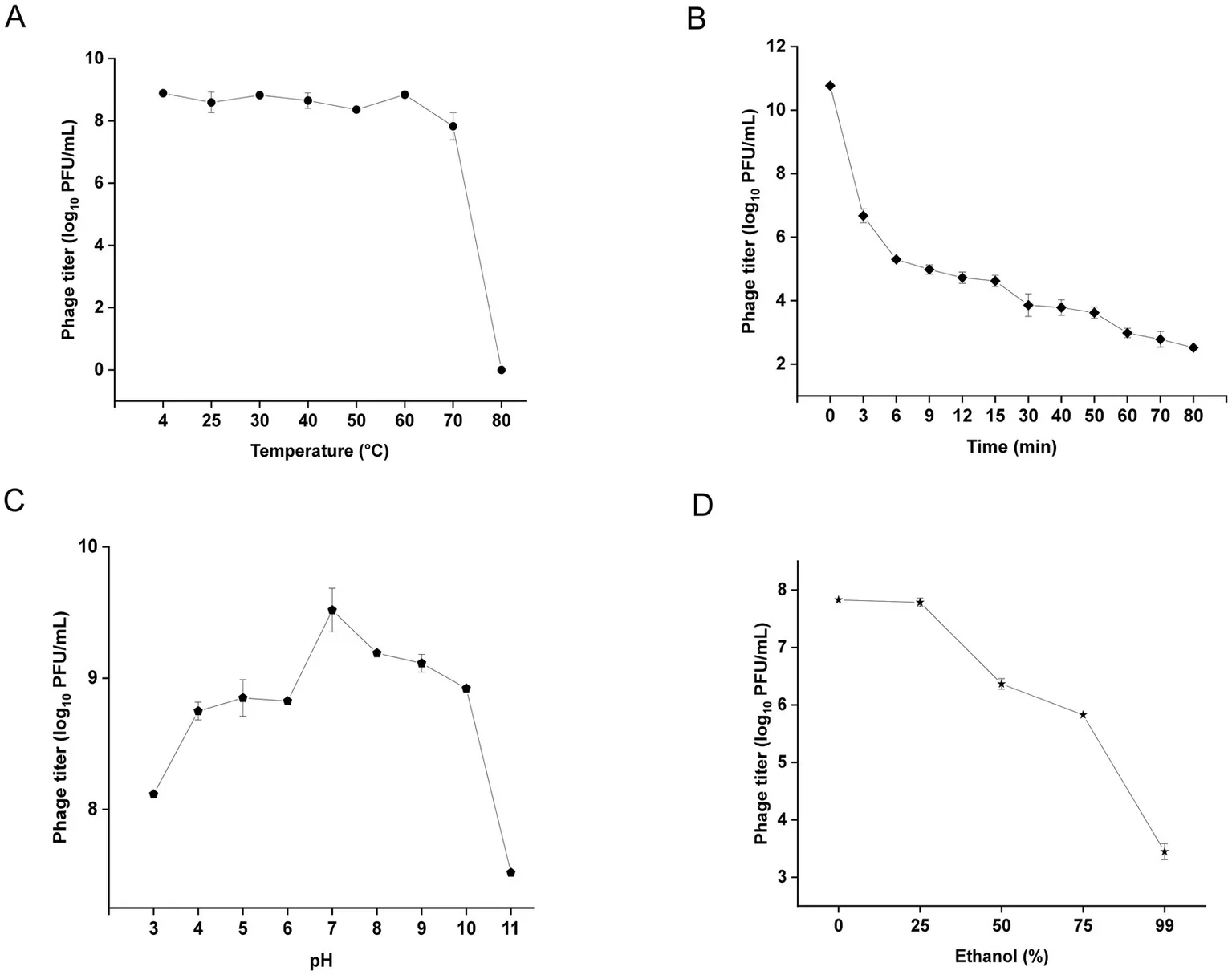
Physicochemical stability of phage WEN7. **(A)** Thermal stability was assessed across a temperature range of 4–80 °C. **(B)** UV tolerance was evaluated over 0–80 min of irradiation exposure. **(C)** pH stability was tested in the range of pH 3–11. **(D)** Ethanol tolerance was determined at concentrations from 0–99%. Error bars represent SD from three independent experiments.

Phage WEN7 was tested for stability under various conditions, including temperature, ultraviolet (UV) irradiation, pH, and ethanol. The phage maintained high titers (10^8^-10^9^ PFU/mL) from 4 °C to 70 °C, but no viable phage was detected at 80 °C (Figure 3A). Under UV exposure, the titer gradually declined over time. Nevertheless, the phage remained detectable even after 80 min, with a titer of around 10^2^ PFU/mL (Figure 3B). The phage was stable from pH 3.0 to pH 10.0, with titers of 10^8^-10^9^ PFU/mL and peak activity at pH 7.0, whereas viability dropped sharply at pH 11.0 (Figure 3C). Ethanol tolerance tests showed that phage WEN7 retained substantial titers at 25, 50, and 75% ethanol (10^8^, 10^6^, and 10^5^ PFU/mL, respectively), while exposure to 99% ethanol reduced the titer to approximately 10^3^ PFU/mL (Figure 3D).

### 3.5 Genome characteristics analysis

Genomic characterization of WEN7 revealed a circular double-stranded DNA molecule of 66,379 bp with a GC content of 55.62%. Ninety-eight open reading frames (ORFs) were predicted, of which 21 were annotated and grouped into four functional categories: structural proteins, DNA replication and recombination, DNA packaging and lysis, and DNA metabolism and modification (Table 3). No virulence factors, antibiotic resistance genes, or lysogeny-associated genes were identified in the genome. The genome sequence of phage WEN7 has been deposited in GenBank under accession number PZ145202.

**Table 3:** Predicted ORFs of phage WEN7.

ORFs	Strand	Start	End	Length (bp)	Encoded protein
1	+	1	594	594	Phage tail fiber
3	+	1,036	3,612	2,577	Phage internal (core) protein
6	+	5,064	5,729	666	Phage baseplate
9	+	8,555	11,449	2,895	Phage baseplate
11	+	11,879	12,541	663	Phage endolysin
13	−	14,007	13,096	912	DNA ligase, phage-associated
14	−	14,616	14,062	555	Phage DNA-binding protein
18	−	18,534	16,975	1,560	Phage DNA helicase
20	−	22,041	18,934	3,108	DNA polymerase III alpha subunit (EC 2.7.7.7)
22	−	23,687	22,671	1,017	3′-phosphatase, 5′-polynucleotide kinase, phage-associated
24	−	24,800	23,883	918	Thymidylate synthase ThyX (EC 2.1.1.148)
27	−	25,489	25,271	219	Phage tail assembly protein
32	−	29,356	28,169	1,188	Phage DNA helicase
39	+	33,839	34,006	168	Phage-associated DNA primase
40	+	34,003	35,070	1,068	Phage-associated DNA primase
42	+	35,337	35,480	144	Phage-associated DNA primase
60	−	43,214	42,528	687	Phage capsid and scaffold
65	+	44,495	45,877	1,383	Phage terminase, large subunit
81	+	54,682	55,518	837	Phage minor capsid protein
86	+	58,474	59,622	1,149	Phage capsid and scaffold
93	+	63,764	64,216	453	Phage tail fiber

**Figure 4:**
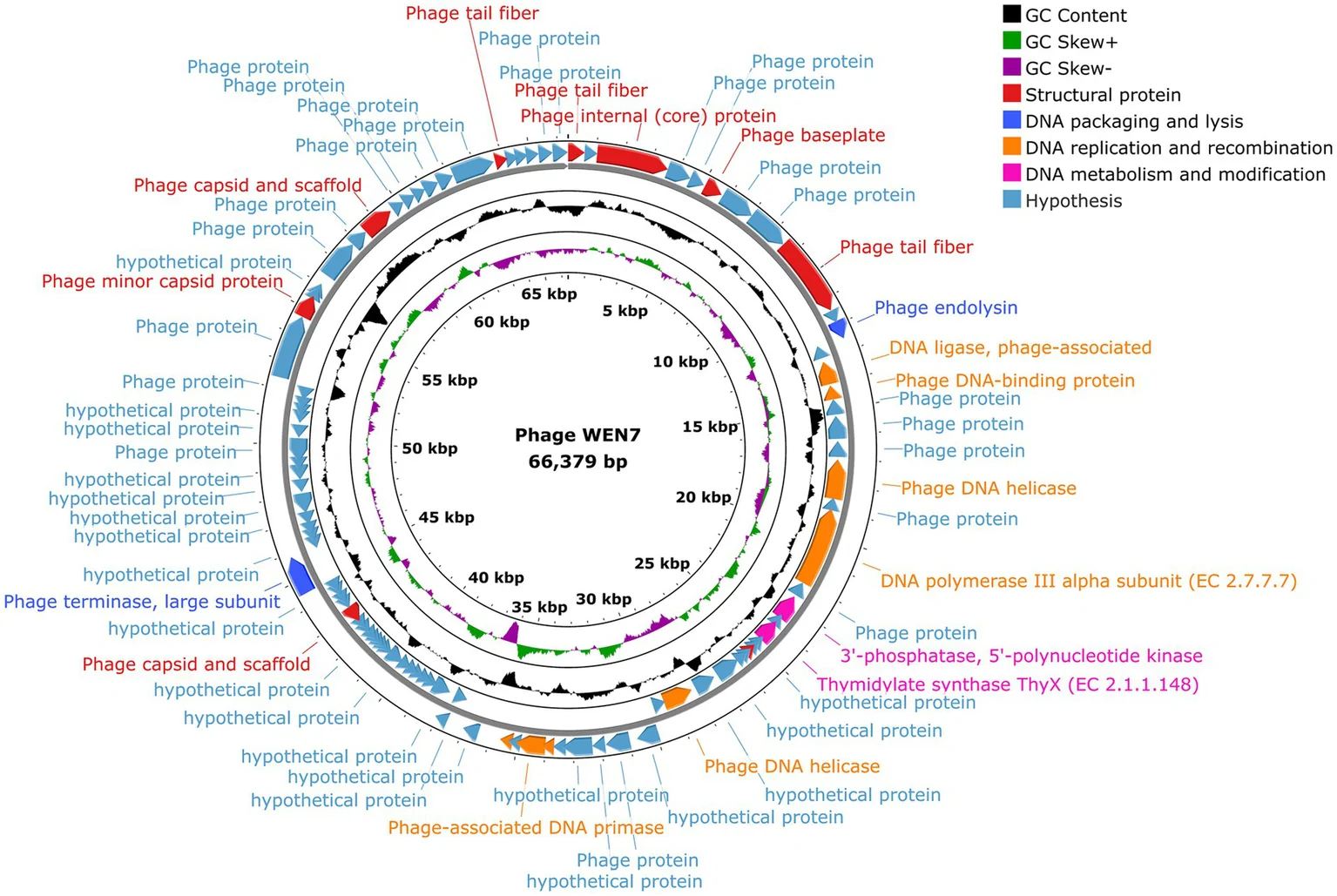
Whole-genome map of phage WEN7. Genome visualization using Proksee revealed a 66,379 bp genome with 98 predicted genes. GC content and GC skew were plotted alongside functional annotations. Annotated phage genes were classified into four functional categories: structural protein genes (red), DNA packaging and lysis (blue), DNA replication and recombination (orange), and DNA metabolism and modification (magenta).

The annotated ORFs fall into the following categories. Structural proteins: genes encoding capsid and scaffold, minor capsid, tail fibers, baseplate, tail assembly, and internal core proteins. These proteins are required for phage particle assembly and structural integrity. DNA replication and recombination proteins: DNA helicase, DNA polymerase III alpha subunit, primase, DNA-binding protein, and DNA ligase. These proteins drive the replication and repair of the phage genome. DNA packaging and lysis genes: terminase large subunit (packages the viral genome into the capsid) and endolysin (degrades the host cell wall to release progeny phages). DNA metabolism and modification genes: 3′-phosphatase/5′-polynucleotide kinase and thymidylate synthase ThyX. These enzymes support nucleotide synthesis and DNA modification, facilitating phage proliferation (Figure 4).

### 3.6 Genome collinearity analysis

**Figure 5:**
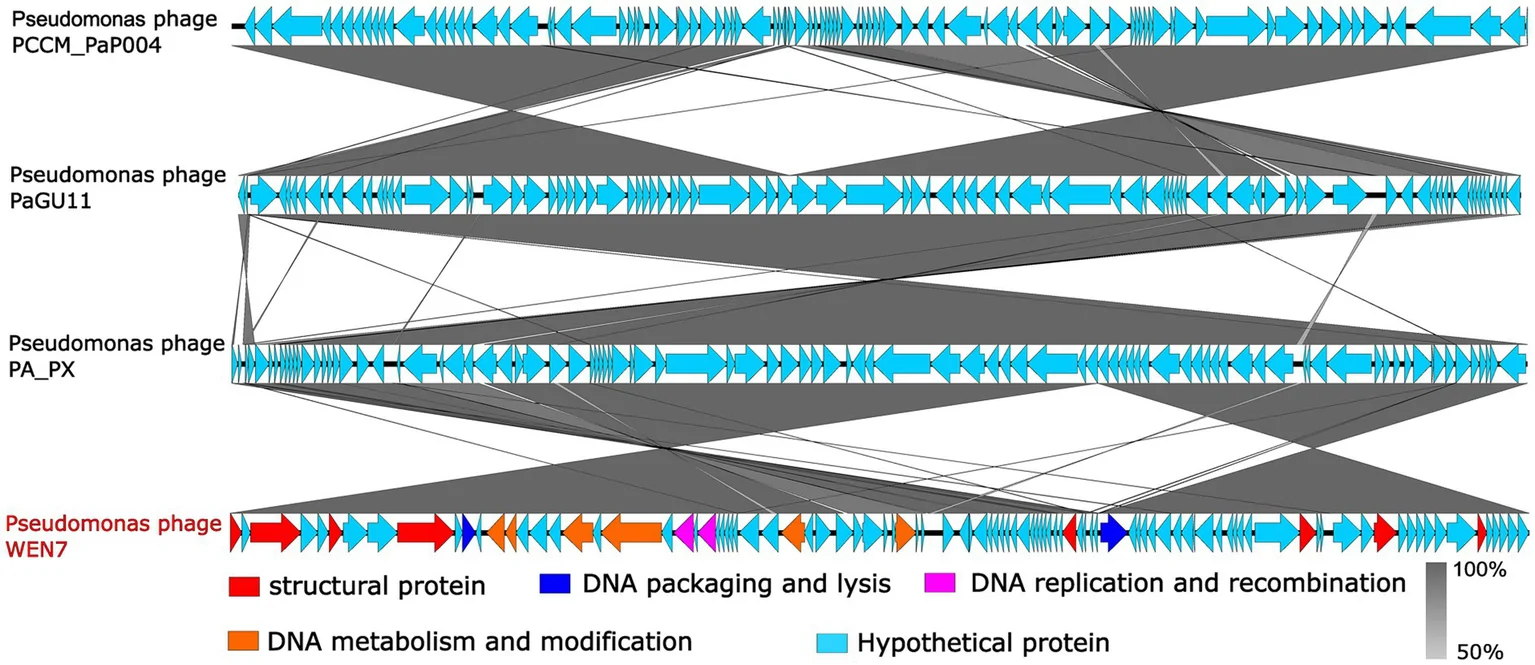
Genome collinearity analysis. Comparative genome analysis of the *Pseudomonas aeruginosa* phages WEN7, PCCM_PaP004, PaGU11, and PA_PX. The transcription direction is indicated by arrows, with arrow colors assigned according to functional categories.

BLAST analysis against the NCBI database revealed that phage WEN7 shared the highest similarity with *Pseudomonas* phage PCCM_PaP004 (accession PQ761513.1), showing 99% genome coverage and 97.20% nucleotide identity (max score 58,818; Supplementary Table S2). Genome collinearity analysis indicated that WEN7 was largely syntenic with the reference *Pseudomonas* phages PCCM_PaP004, PaGU11, and PA_PX. Core functional modules, including DNA replication components (e.g., DNA polymerase and DNA ligase) and structural proteins (e.g., tail proteins), were highly conserved. However, the region encoding the terminase and capsid proteins showed lower homology and disrupted synteny compared with the reference phages (Figure 5).

### 3.7 Phylogenetic tree and ANI analysis

**Figure 6:**
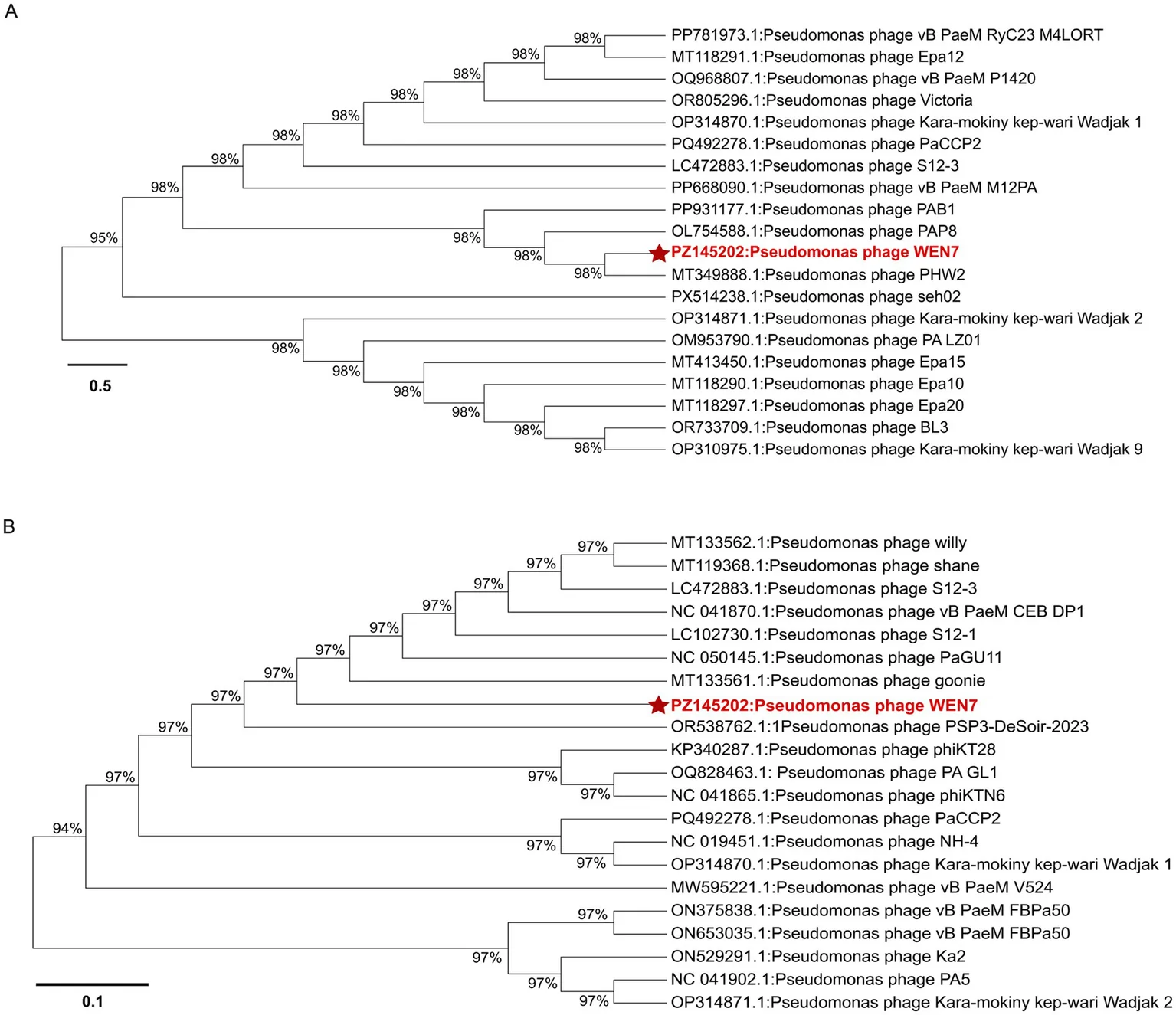
Phylogenetic trees of phage WEN7. **(A)** Phylogenetic tree based on capsid and scaffold proteins of selected phages. **(B)** Phylogenetic tree based on terminase large subunit sequences of selected phages. Both trees were constructed using the neighbor-joining method with 1,000 bootstrap replicates. Phage WEN7 is highlighted in red with an asterisk.

Phylogenetic trees were constructed based on the capsid-scaffold protein and the terminase large subunit. In the capsid protein tree, phage WEN7 clustered with *Pseudomonas* phages PAP8 (OL754588.1) and PHW2 (MT349888.1) with 98% bootstrap support. This clade was nested within a larger group of *Pseudomonas* phages (bootstrap support 95%) (Figure 6A). In the terminase tree, WEN7 grouped with *Pseudomonas* phage goonie (MT133561.1) in a clade with 97% bootstrap support (Figure 6B).

**Figure 7:**
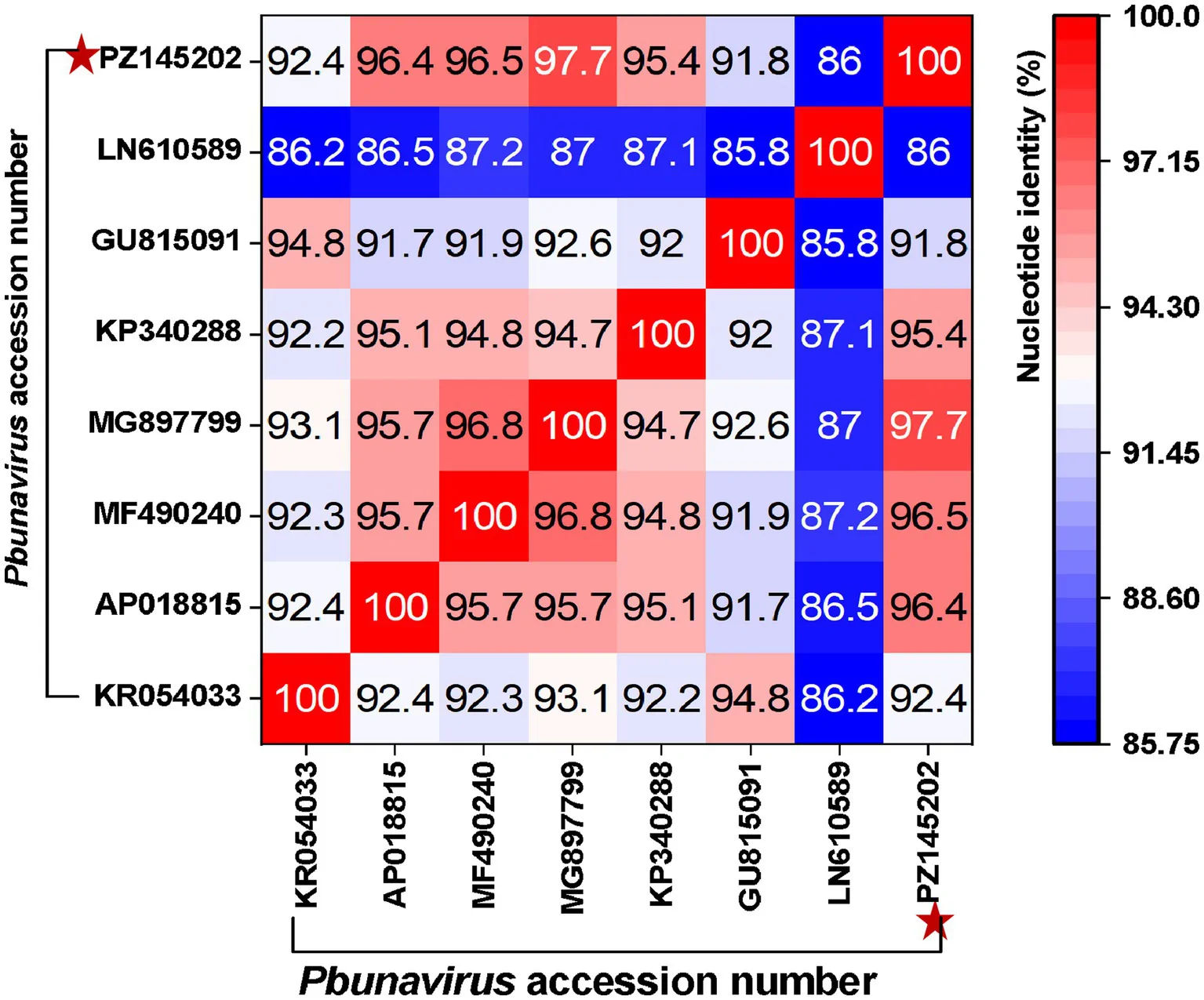
ANI analysis of phage WEN7 and related phages. The heatmap shows the nucleotide identity of WEN7 with the other seven phages of *Pbunavirus*. The diagonal elements represent 100% identity (self-comparison). Color intensity corresponds to the percentage of nucleotide identity, with red indicating higher identity and blue indicating lower identity.

Average nucleotide identity (ANI) was calculated between phage WEN7 and seven reference *Pseudomonas* phages of the genus *Pbunavirus*. The highest ANI (97.7%) was observed with vB_PaeM_LS1 (MG897799). Phages vB_PaeM_E217 (MF490240), PaGU11 (AP018815), and phiKTN6 (KP340288) showed ANI values near 95%, while vB_PaeM_C1-14_Ab28 (LN610589) had values below 90% (Figure 7).

### 3.8 Bactericidal activity of phage WEN7

**Figure 8:**
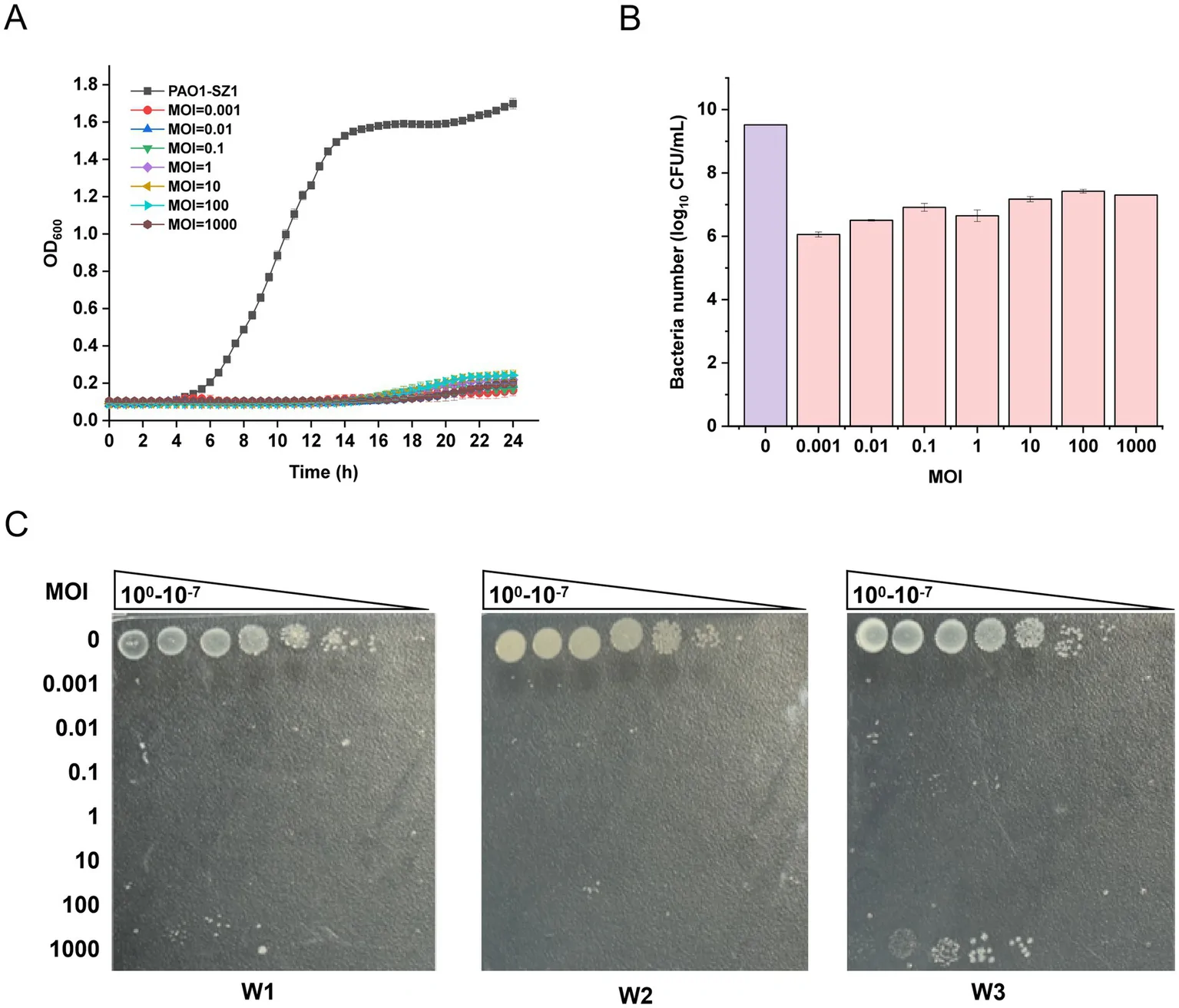
Bactericidal activity of phage WEN7 at different MOIs. **(A)** Growth curve in LB medium (OD_600_, 24 h). **(B)** Bactericidal effect in milk (log_10_ CFU/mL). **(C)** Surviving host bacteria in wastewater after co-incubation with phage at different MOIs. W1, W2, and W3 represent wastewater from the Shenzhen Institute of Translational Medicine, Shenzhen Second People’s Hospital, and Shenzhen Children’s Hospital, respectively. In all cases, untreated bacteria (no phage, MOI = 0) served as the control group. Error bars represent SD from three independent experiments.

The bactericidal activity of phage WEN7 was evaluated under laboratory and environmental conditions. In LB medium, the control culture (PAO1-SZ1 without phage) reached an OD_600_ of 1.6 after 24 h at 37 °C. In contrast, cultures treated with phage at MOIs ranging from 0.001 to 1,000 remained below 0.3 throughout the incubation period (Figure 8A). Thus, under laboratory conditions, phage WEN7 showed strong bactericidal activity across all MOIs tested, with no apparent difference between low and high multiplicities. In pasteurized milk, the host strain in the absence of phage (MOI = 0) reached 10^9^ CFU/mL. In phage-treated samples, bacterial counts were at least 10-fold lower than the control. The strongest effect was observed at an MOI of 0.001, where viable counts dropped by 1,000-fold (Figure 8B). In hospital wastewater, the host strain reached 10^10^ CFU/mL in the absence of phage. At MOIs of 0.001 and 100, few viable bacteria were detected. However, at an MOI of 1,000, colonies were still visible, particularly in the sample from Shenzhen Children’s Hospital (Figure 8C).

## 4 Discussion

Multidrug-resistant *P. aeruginosa* poses a major therapeutic challenge, and the declining efficacy of conventional antibiotics has fuelled renewed interest in phage therapy. While the model strain PAO1 is widely used for evaluating new antimicrobials, its laboratory evolution and the emergence of resistant derivatives, together with the limited size of the current phage library, underscore the need for continuous isolation and characterization of novel phages from the environment.

In this study, we isolated a lytic phage, WEN7, using a multidrug-resistant PAO1 derivative (PAO1-SZ1) as the host. The TEM image showed both extended and contracted tail—classic myovirus morphology (Comeau et al., 2012). Under the latest ICTV classification, WEN7 belongs to the class Caudoviricetes (Turner et al., 2023). The burst size of WEN7 (165 PFU/cell) exceeds those of many recently described *P. aeruginosa* phages, such as PSA-KC1 (85 PFU/cell) (Kurt et al., 2025) and PaeP_Ls (50 PFU/cell) (Luo et al., 2026), and is comparable to that of Pa_WF01 (154 PFU/cell) (Sun et al., 2025). While some phages, including Pae01 (10^6^ PFU/cell) (Shi et al., 2024), exhibit substantially higher burst sizes, extremely high replication rates may not always correlate with improved therapeutic outcomes, as factors such as stability and host range often play equally critical roles. Phage WEN7 demonstrated remarkable stability across a wide range of physical and chemical conditions. It remained active up to 70 °C and across pH 3.0–11.0, outperforming many previously reported *P. aeruginosa* phages, including Pae01 and vB_PaeM_PS3 (Abdelghafar et al., 2023), which are inactivated at approximately 60–70 °C, and vB_PaeP_GZMU_A1002 (Fu et al., 2026), which shows a narrower pH tolerance. Additionally, WEN7 retained detectable activity after 80 min of UV exposure and remained viable at 75% ethanol—conditions relevant to routine disinfection protocols. These properties suggest that WEN7 could be readily incorporated into existing physical or chemical disinfection protocols without requiring special handling.

Genome sequencing confirmed that WEN7 carries a circular double-stranded DNA of 66,379 bp with a GC content of 55.62%. The annotation rate was relatively low, which is not uncommon for newly isolated environmental phages and likely reflects the presence of many hypothetical proteins with unknown functions. Functional annotation identified genes for capsid and scaffold proteins, tail fibers, baseplate, and endolysin—consistent with the observed myovirus morphology and lytic lifestyle. The presence of DNA polymerase III alpha subunit, helicase, and DNA-binding protein points to a replication system with limited dependence on the host, which may contribute to the observed rapid proliferation (Petrov et al., 2010). Importantly, no antibiotic resistance genes, virulence factors, or lysogeny-associated genes were detected, and no known CRISPR antagonism elements were found (Mendoza et al., 2020; Hobbs et al., 2022). These genomic features support the biosafety of WEN7 and its suitability for therapeutic applications, although the functional roles of the 77 hypothetical ORFs warrant future investigation.

Host range analysis showed that WEN7 lysed 66.7% (20/30) of the tested *P. aeruginosa strains*, a rate that falls within the upper-mid range compared with other lytic phages. For instance, Zhang et al. reported that five lytic phages lysed 29–76% of 246 clinical strains (Zhang et al., 2025), while Fei et al. described phage HZ2201 with a lysis rate of 78.38% (Fei et al., 2023). Notably, all susceptible clinical isolates carried at least one carbapenem resistance determinant, and several of them (e.g., PA24, PA26, PA29) were resistant to eight or more antibiotics spanning three major classes (cephalosporins, penicillins, and fluoroquinolones). These multidrug-resistant (MDR) strains are among the most challenging pathogens in current clinical practice. In the absence of rapid, precise phage-matching diagnostics, broad lytic activity significantly enhances the applicability and success rate of phage therapy (Abedon et al., 2011). The ability of WEN7 to infect such highly resistant strains, without prior matching, highlights its value as a broad-spectrum candidate, either as a stand-alone therapeutic or as a component of a phage cocktail.

Finally, we evaluated the bactericidal activity of WEN7 in two real-world matrices: pasteurized milk and hospital wastewater. In milk, WEN7 demonstrated significant bactericidal activity across a wide range of MOIs, with higher killing efficiency observed at lower MOIs, highlighting its strong potential for application in food safety. In hospital wastewater samples, near-complete bacterial clearance was achieved at MOIs ranging from 0.001 to 100, but the highest MOI (1000) paradoxically resulted in poorer killing, particularly in one of the samples (Shenzhen Children’s Hospital). This observation suggests that very high MOIs may impose stronger selective pressure, potentially accelerating the emergence of resistant mutants (Chan et al., 2016). The phenomenon of high killing efficiency at low MOIs has also been reported for other *P. aeruginosa* phages, such as vB_PaeM_QR7, which reduced bacterial loads by 5.40 ± 0.12 log_10_ CFU/mL at an extremely low MOI of 1 × 10^−5^ (Pu et al., 2026). Similarly, phage vB_Ps_ZCPS13 exhibited optimal bactericidal activity at MOI = 0.1 (Mohamed et al., 2025). These findings underscore the importance of optimizing MOI for each specific application context rather than simply maximizing phage dose. Given the dual challenge of multidrug resistance and phage resistance, future work should explore phage-antibiotic combinations to further suppress resistance evolution and enhance therapeutic persistence. In addition, while the use of sterile wastewater allowed for a controlled evaluation, testing phage efficacy in non-sterile environmental samples will be important to better reflect real-world conditions and interactions with resident microbial communities.

Although WEN7 shows promising therapeutic potential, several key directions remain unaddressed. Its capacity to prevent and eliminate biofilms—critical for chronic *P. aeruginosa* infections—has not been assessed. Moreover, activity in non-sterile environmental samples requires systematic evaluation, as native microbial communities may compromise efficacy. Future work will focus on the functional roles of the 77 hypothetical proteins, host recognition mechanisms, and evasion of bacterial defense systems. Ultimately, *in vivo* safety and efficacy studies, together with phage-antibiotic synergy assessments, are essential next steps toward clinical translation.

## 5 Conclusion

Here we report the isolation and characterization of WEN7, a lytic phage obtained from hospital wastewater using a multidrug-resistant *P. aeruginosa* strain as the host. WEN7 displays a broad host range and remarkable stability across diverse temperatures and pH conditions. Morphological and genomic analyses place it within the class Caudoviricetes. Its genome lacks known antibiotic resistance or virulence genes, supporting its safety profile. Notably, WEN7 exhibited strong bactericidal activity in milk and hospital wastewater, even at low multiplicities of infection. Together, these features, including broad host range, environmental robustness, genomic safety, and efficacy in complex matrices, position WEN7 as a promising candidate for phage therapy against multidrug-resistant *P. aeruginosa* infections.

## Data Availability

The datasets presented in this study can be found in online repositories. The names of the repository/repositories and accession number(s) can be found at: https://www.ncbi.nlm.nih.gov/genbank/, PZ145202.
