# Supplementary material for: Characterization of a newly isolated broad-host-range lytic phage WEN7 against multidrug-resistant clinical Pseudomonas aeruginosa strains
Source: Front Microbiol. 2026 Aug 12;17:1861197. doi: 10.3389/fmicb.2026.1861197 (PMC13506937; doi:10.3389/fmicb.2026.1861197)
Supplement: Supplementary file 1 [file Supplementary_file_1.DOCX]

**Supplementary Material**

**Supplementary Figure**

**Supplementary Figure 1.** Host range of phage WEN7*.* Plaques formed by WEN7 on lawns of different tested strains*.*

**Supplementary Tables**

**Supplementary Table 1: Antibiotic resistance of clinical isolates of *P. aeruginosa.***

| **Strains** | **Meropenem** | **Imipenem** | **Faropenem** | **Ertapenem** | | **Ceftazidime** | | **Cefoperazone** | **Cefepime** | **Piperacillin** | **Ticarcillin** | **Aztreonam** | **Amikacin** | **Tobramycin** | **Ciprofloxacin** | **Levofloxacin** | **Colistin** |
| --- | --- | --- | --- | --- | --- | --- | --- | --- | --- | --- | --- | --- | --- | --- | --- | --- | --- |
| PA-1 | S | S | I | I |  | |  | |  |  |  |  |  |  |  |  |  |
| PA-3 | S | I | R | R |  | |  | |  |  |  |  |  |  |  |  |  |
| PA-4 | S | R | R | R |  | |  | |  |  |  |  |  |  |  |  |  |
| PA-5 | S | S | R | R |  | |  | |  |  |  |  |  |  |  |  |  |
| PA-6 | S | S | R | S |  | |  | |  |  |  |  |  |  |  |  |  |
| PA-7 | S | S | S | R |  | |  | |  |  |  |  |  |  |  |  |  |
| PA-8 | S | R | R | S |  | |  | |  |  |  |  |  |  |  |  |  |
| PA-9 | S | S | S | S |  | |  | |  |  |  |  |  |  |  |  |  |
| PA-10 | S | I | R | S |  | |  | |  |  |  |  |  |  |  |  |  |
| PA-11 | S | S | R | R |  | |  | |  |  |  |  |  |  |  |  |  |
| PA-12 | S | I | R | S |  | |  | |  |  |  |  |  |  |  |  |  |
| PA-13 | S | S | R | R |  | |  | |  |  |  |  |  |  |  |  |  |
| PA-14 | S | S | R | R |  | |  | |  |  |  |  |  |  |  |  |  |
| PA-15 | S | S | R | S |  | |  | |  |  |  |  |  |  |  |  |  |
| PA-16 | S | S | R | R |  | |  | |  |  |  |  |  |  |  |  |  |
| PA-17 | S | S | R | R |  | |  | |  |  |  |  |  |  |  |  |  |
| PA-18 | S | S | R | R |  | |  | |  |  |  |  |  |  |  |  |  |
| PA-19 | S | S | R | R |  | |  | |  |  |  |  |  |  |  |  |  |
| PA-20 | S | S | R | R |  | |  | |  |  |  |  |  |  |  |  |  |
| PA-21 | S | I | R | R |  | |  | |  |  |  |  |  |  |  |  |  |
| PA-22 | S | I | R | R |  | |  | |  |  |  |  |  |  |  |  |  |
| PA-23 | R | R |  |  | R | | R | | R | R | R | R | S | S | S | S | S |
| PA-24 | R | R |  |  | R | | S | | R | S | R | R | S | S | R | R | I |
| PA-25 | S | R |  |  | S | | S | | S | S | S | S | S | S | R | R | I |
| PA-26 | R | R |  |  | I | | R | | S | I | R | R | S | S | S | R | I |
| PA-27 | I | R |  |  | S | | S | | S | S | S | S | S | S | S | S | I |
| PA-28 | R | R |  |  | S | | S | | S | S | I | S | S | S | S | S | I |
| PA-29 | R | R |  |  | I | | R | | I |  | R | R | R | R | R | R | I |
| PA-30 | R | R |  |  | R | | I | | S |  | R | R | S | S | R | R | I |

**Note: S = susceptible, I = intermediate, R = resistant. Blank cells indicate that the antibiotic was not tested against the corresponding strain.**

**Supplementary Table 2:** **Genomic comparison of phage WEN7 with closely related *Pseudomonas* phages.**

| **Phage** | **Max Score** | **Coverage (%)** | **Identity (%)** | **Length (bp)** | **Accession** |
| --- | --- | --- | --- | --- | --- |
| *Pseudomonas* phage PCCM_PaP004 | 58818 | 99 | 97.20 | 66227 | PQ761513.1 |
| *Pseudomonas* phage PaGU11 | 55335 | 98 | 98.54 | 65554 | NC 050145.1 |
| *Pseudomonas* phage PA_PX | 54713 | 99 | 98.18 | 66201 | PV189959.1 |
| *Pseudomonas* phage Kara-mokiny_kep-wari_Wadjak_1 | 54685 | 99 | 98.77 | 67075 | OP314870.1 |
| *Pseudomonas* phage vB3530 | 54654 | 98 | 98.75 | 66392 | OR075999.1 |

**Note: Only the five most similar phages are shown.**
